# Possible role of pre-vaccination T-lymphocyte subpopulations in the antibody response to COVID-19 vaccines in children undergoing chemotherapy

**DOI:** 10.3389/fimmu.2026.1728845

**Published:** 2026-02-05

**Authors:** Csaba Péterfia, Zsolt I. Komlósi, Zoltán Pós, Nikolett Lupsa, Nóra Fekete, Katalin Böröcz, Timea Dergez, Evelin A. Leibinger, Noémi Benedek, Ágnes Vojcek, Bence Horváth, Vita Vertike, Krisztina Csanádi, Péter Hauser, Lilla Györgyi Tiszlavicz, Dániel János Erdélyi, Edit Ágota Brückner, Sándor Szabó, Nikolett Jusztina Beniczky, Timea Berki, Gábor Ottóffy

**Affiliations:** 1 Division of Pediatric Hematology and Oncology, Department of Pediatrics, University of Pécs Medical School, Pécs, Hungary; 2 Department of Genetics, Cell- and Immunobiology, Semmelweis University, Budapest, Hungary; 3 Department of Immunology and Biotechnology, Medical School, University of Pécs, Pécs, Hungary; 4 Institute of Bioanalysis, Medical School, University of Pecs, Pecs, Hungary; 5 Hemato-Oncology Unit, Heim Pal Children’s Hospital, Budapest, Hungary; 6 Haematology/Oncology and Pediatric Bone Marrow Transplantation Unit, Child Health Centre, Borsod-Abauj-Zemplen County Hospital, Miskolc, Hungary; 7 Faculty of Health Sciences, University of Miskolc, Miskolc, Hungary; 8 Department of Pediatrics, University of Szeged, Szeged, Hungary; 9 Pediatric Centre, Semmelweis University, Budapest, Hungary

**Keywords:** CD3+CD56+ T-cells, CD4+ MAIT cells, COVID-19, immune response, immunosuppression, lymphocyte subpopulations, pediatric oncology, SARS-CoV-2 vaccination

## Abstract

**Background:**

In a previous study, we found a possible connection between pre-vaccination CD3+CD56+ T cells and seroresponse to influenza vaccination in immunosuppressed patients. Decreased circulating CD3+CD56+ T cells have been described in severe COVID-19, but their role in vaccination is unknown. This study evaluated the humoral immune response after SARS-CoV-2 vaccination in children with cancer following two doses of the BNT162b2 mRNA vaccine. We investigated the relationship between cellular immunity (including CD3+CD56+ T cells) and the post-vaccination antibody response.

**Methods:**

A multicenter, prospective cohort study was completed by recruiting patients receiving chemotherapy and healthy controls, who received two doses of the BNT162b2 mRNA vaccine. Flow cytometric analysis of peripheral blood lymphocyte subpopulations was performed before vaccination; serum anti-SARS-CoV-2 IgG was measured before vaccination and 21–28 days after the second vaccination. We evaluated the relationship between various cellular parameters before vaccination and antibody response.

**Results:**

Serological response was assessed in 20 oncology patients and 13 healthy controls. The seroconversion rate was 55% among oncology patients and 92.3% among healthy controls (p = 0.023). Geometric mean fold increase (GMFI) of the titers was 6.69 and 41.64 (p = 0.011), respectively. Flow cytometric analysis revealed a significantly higher seroconversion rate in patients with higher baseline CD3+CD56+ T cell (p = 0.044) and CD56+ NK (p = 0.038) cell counts. Based on GMFI, we found a positive association between a greater antibody response and higher baseline CD4+ (p = 0.007), CD4+CD3+CD56+ (p = 0.019), and CD4+ MAIT (p = 0.010) cell counts, as well as a higher CD4/CD8 ratio (p = 0.029).

**Conclusion:**

Our study suggests that an adequate humoral immune response can be induced by the SARS-CoV-2 mRNA vaccine in pediatric oncology patients. We explored for the first time the possible association between pre-vaccination T lymphocyte subpopulations (CD3+CD56+, CD56+ NK, CD4+, CD4+CD3+CD56+ cells) and the antibody response to the COVID-19 vaccine. We have similar observations as previously reported with influenza vaccination, suggesting that CD3+CD56+ T cells may be involved in the immune response to SARS-CoV-2 vaccines. We highlight the connection between pre-vaccination CD4+ MAIT cell populations and the antibody response.

## Introduction

Coronavirus disease 2019 (COVID-19), which can manifest as a severe respiratory disease, is caused by severe acute respiratory syndrome coronavirus 2 (SARS-CoV-2). COVID-19 first emerged in the Wuhan region of China in late 2019 and soon became a pandemic.

By 2025, over 777 million confirmed cases and more than 7 million deaths have been reported worldwide (1). In children, the disease is milder, often asymptomatic, and rarely severe, unlike in adults. Deaths are rare and occur mainly in children with an underlying disease (2).

In children with cancer, severe morbidity and mortality associated with COVID-19 are more frequent than in the general pediatric population. Delaying oncological treatment due to an infection reduces the chance of curing the underlying disease (3, 4). Therefore, prevention, including vaccination, is essential in this patient group.

Studies have demonstrated the safety and efficacy of available COVID-19 vaccines in adult cancer patients, although the serological response is lower than in healthy adults (5–8). However, at the time of our study, research on the efficacy of the vaccine in pediatric oncology patients, particularly those under 12 years of age, was still scarce (9–11).

Therefore, it is particularly important to understand the immunological mechanisms underlying a successful immune response in patients with low white blood cell counts due to undergoing oncological treatment.

Studies on influenza vaccination have revealed several associations between cellular immunity in children treated for cancer and response to vaccination, such as a positive association between baseline higher lymphocyte, CD3+, CD4+, and CD19+ values and serological response (12–17). A similar observation with COVID-19 vaccination has been described only in adult patients (18), only in connection with the positive predictive role of higher total lymphocyte counts and higher CD4/CD8 ratios. The activation of unconventional T cells (such as γδ T cells, iNKT cells, and MAIT cells) has been related to the course of COVID-19, with lower numbers circulating in severe infections (19–21). Furthermore, the circulating number of MAIT cells prior to vaccination appears to be associated with the humoral immune response following BNT162b2 mRNA vaccination (22).

In a previous study (16), we found a positive association between pre-vaccination CD3+CD56+ T cell count above the age-specific predicted values and the antibody response rate after influenza vaccination in children receiving chemotherapy. These observations have drawn our attention to the role of the natural immune system in the immune response to vaccines. A decreased proportion of circulating CD56+ T cells in patients hospitalized for COVID-19 was associated with more severe outcomes (23, 24) and a higher mortality rate (25), highlighting the role of these cells in COVID-19 infection.

This multicentric study aimed to assess the humoral immune response after SARS-CoV-2 vaccination in 5-18-year-old oncology patients and healthy children following two doses of the BNT162b2 mRNA COVID-19 vaccine. We investigated the relationship between the pre-vaccination proportions and absolute numbers of T lymphocyte subpopulations involved in adaptive and innate immunity and the post-vaccination antibody response. In addition, we aimed to collect data on the proportions and absolute cell counts of individual T lymphocyte subpopulations in specific age groups of healthy children.

## Materials and methods

### Patient characteristics

This study was a multicenter, prospective cohort study conducted by the Pediatric Oncology Unit of the University of Pecs Medical School. Between October 2021 and June 2022, we enrolled twenty-one consecutive patients aged 5–18 years receiving chemotherapy for various types of cancer at five oncology centers in Hungary and nineteen healthy children as a control group. All participants received two doses of the mRNA vaccine BNT162b2 (Comirnaty, BioNTech/Pfizer). All oncology patients had received chemotherapy within one month before vaccination, and all continued to receive scheduled chemotherapy afterward. In patients receiving maintenance treatment, the vaccine was administered without discontinuing therapy. During intensive treatment, vaccination was scheduled for all patients 2–3 days before the subsequent cytostatic treatment, per the recommendations of the Hungarian National Healthcare Advisory Board. The time between the last chemotherapy block and vaccination and blood sampling was at least 2 weeks for each patient. The absolute neutrophil count was over 0.5 G/L at that time. There was no patient with corticosteroid exposure. We excluded all children who acquired COVID-19 infection immediately before vaccination and those who had a general contraindication to vaccination.

**Figure 1:**
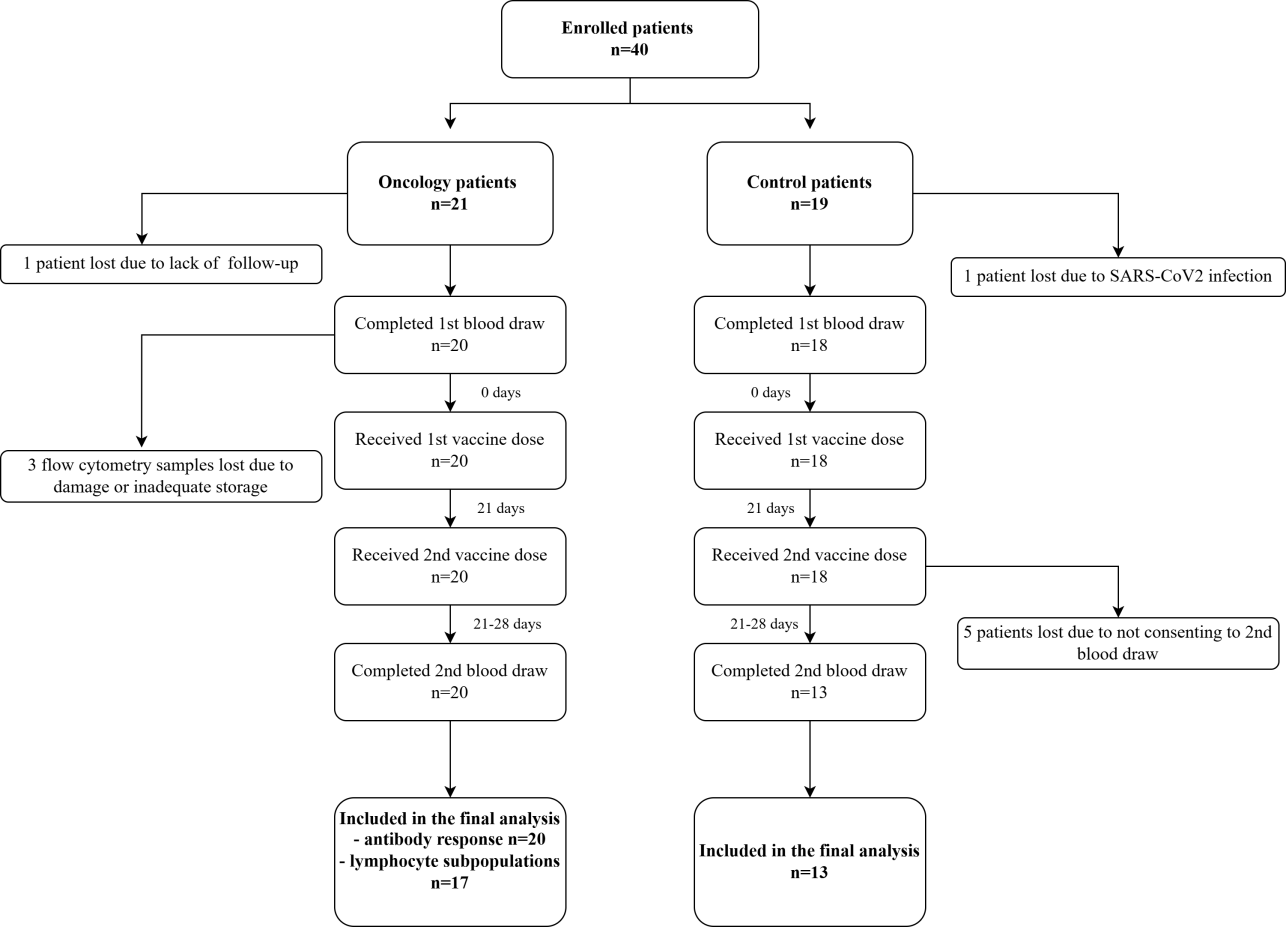
Flowchart of participants.

The study included 40 children: 21 oncology patients and 19 healthy controls. One oncology patient was lost to follow-up, and one healthy participant was excluded due to serology results suggesting a recent COVID-19 infection immediately prior to the first sampling. Five children from the control group did not consent to the second sampling (**Figure 1**).

Before the study began, we conducted *a priori* sample size estimation based on a previously published influenza vaccine study involving a similar pediatric oncology cohort. The study aimed to achieve 80% statistical power. However, the actual recruitment of eligible oncology patients for the study was substantially lower than expected due to clinical availability and a decline in vaccination uptake over time. The worldwide decline in vaccination uptake may also have contributed to this, and several authors have investigated the reasons for it (26, 27). **Supplementary Table 1** presents the data for the twenty oncology patients in the study by sex, age, tumor type, and treatment type.

Serological responses of 33 children, including 20 oncology patients and 13 healthy controls, were analyzed (**Table 1**). The age and sex distributions of the oncology patient and healthy control populations were matched. Due to damage or inadequate transportation of three patients’ samples, we were able to analyze the flow cytometry results of only 17 oncology patients.

**Table 1:** Baseline characteristics of the study population.

Characteristic	Oncology patients (n=20)	Healthy controls (n=13)	P-value
Age (years), mean (SD)	11.96 (± 4.47)	12.82 (± 2.72)	0.456
Age 5–11 years/0.1 ml vaccine, n (%)	10/20 (50)	4/13 (30.8)	0.414
Age 12–18 years/0.3 ml vaccine, n (%)	10/20 (50)	9/13 (69.2)	
Sex (M/F), n (%)	11/9 (55/45)	5/8 (38.5/61.5)	0.353
Cancer type (hematological/solid), n (%)	10/10 (50/50)	–	
Chemotherapy (intensive/maintenance), n (%)	9/11 (45/55)	–	

SD, standard deviation; M, male; F, female; n, number of participants.

### Study design, vaccine, and schedule

Written informed consent was obtained from the participating children and their parents. The European Medicines Agency (EMA) has authorized the mRNA vaccine we used (Comirnaty, BNT162b2, Pfizer-BioNTech) (28).

The study was reviewed by the Scientific and Research Ethics Committee of the Medical Research Council and approved by the National Centre for Public Health (File No 51296-8/2021/ECIG, and 74461-5/2021/ECIG). It was conducted in accordance with the Declaration of Helsinki and the guidelines of Good Clinical Practices.

Pfizer-BioNTech’s COVID-19 mRNA vaccine was administered in a carefully planned manner. It was given in two doses 21 days apart, as recommended by the EMA (28). The vaccine was administered as an intramuscular injection, with 30 µg for 12–18 year old children and 10 µg for 5–11 year old children, ensuring the correct dosage for each age group.

### Data collection, blood sample collection, and serological analysis

Before vaccination, demographic data, health data (type of tumor, type of treatment during vaccination, date of vaccination), and health data related to COVID-19 infection were recorded from the electronic health database.

Two blood samples were collected from the participants: one on the day of vaccination, just before administration of the vaccine, and the other 42–49 days after the first vaccination (21–28 days after the second vaccination). Blood was collected from a central venous catheter or via peripheral venipuncture. Blood was drawn for blood count (2 ml, EDTA), serology (4 ml, native), and flow cytometry (6 ml, Na-citrate BD Vacutainer^®^ CPT™). Routine blood cell counts were measured at the Central Laboratory of the University of Pécs, Medical School. Native tubes were centrifuged (2500g, 10 min, room temperature), and serum samples were collected and stored at –80°C until serology analyses. Anti-SARS-CoV-2 IgG levels were measured at the Department of Immunology and Biotechnology, University of Pécs, Medical School, by using an enzyme-linked immunosorbent assay (EUROIMMUN Anti-SARS-CoV-2 QuantiVac IgG) (29).

The geometric mean IgG antibody titer (GMT), GMT fold increase (GMFI: the GMT ratio of the post-vaccination titer to the pre-vaccination titer), and seroconversion rate (defined as a ≥4-fold increase in titer from baseline or a change from seronegative to seropositive status) were calculated.

### Multiparametric flow cytometry analysis of T lymphocyte subsets

The 6 ml Na-citrate BD Vacutainer CPT tube was centrifuged within two hours of blood collection, according to the manufacturer’s instructions. The participating centers sent the obtained PBMC (Peripheral Blood Mononuclear Cells) samples to the Department of Genetics, Cell, and Immunobiology, Semmelweis University within 24 hours. The following antibodies were used for flow cytometric measurements: FITC anti-human CD8 (SK1), PE anti-human TCR Vα7.2 (3C10), PerCP/Cy5.5 anti-human CD4 (RPA-T4), PE/Cy7 anti-human CD56 (HCD56), APC/Cy7 anti-human CD3 (UCHT1), Brilliant Violet 421 anti-human TCR γ/δ (B1), Brilliant Violet 711 anti-human TCR Vα24- Jα18 (6B11); all from Biolegend (San Diego, California, USA). Flow cytometric data were acquired on the same instrument (CytoFLEX S, Beckman Coulter, Brea, California, USA) with the same settings. All samples were analyzed in Kaluza software (Beckman Coulter, Brea, California, USA) by the same researcher (Z.I. Komlósi), keeping the same gating strategy to avoid inter-individual differences in adjusting the gates to each sample. Approximately 2 million cells were acquired from each sample to obtain sufficient resolution for the analysis of rare cell populations. Gating strategies are presented in **Supplementary Figure 1**.

### Statistical analysis

Statistical analysis was performed using IBM SPSS Statistics version 28 to evaluate the laboratory parameters. An independent samples t-test was applied to compare continuous variables between two groups. Results are presented as mean ± standard deviation for the patient and control groups. When three groups were compared based on diagnosis (healthy, hematological, and solid tumor), analysis was conducted using one-way ANOVA followed by Tukey’s *post hoc* test.

For associations between categorical variables, frequency data were analyzed using the χ² test. When the assumptions of this test were not met, Fisher’s exact test was applied instead.

The study aimed to investigate correlations between various cellular parameters before vaccination and the serological response (GMT, GMFI, and seroconversion rates). Pearson’s correlation coefficient was used to assess associations within the oncology group and the healthy control group.

Because of the relatively small sample size and the large number of cellular parameters, we predefined a limited set of primary hypotheses focusing on total lymphocyte counts, CD4^+^ T cells, the CD4/CD8 ratio, CD3^+^CD56^+^ cells, and CD4^+^ MAIT cells. The primary outcomes were predefined based on biological and clinical considerations. For these outcomes, nominal p-values are reported. No formal adjustment for multiple testing was applied; therefore, p-values should be interpreted descriptively, with consideration of effect sizes and consistency of direction rather than strict statistical significance. For these primary variables, in addition to parametric tests (t-tests and Pearson’s correlations on log_10_-transformed titers), we also performed nonparametric sensitivity analyses (Mann–Whitney U tests and Spearman’s rank correlations). Other analyses were considered exploratory. The assumptions of normality for continuous variables were evaluated using Shapiro–Wilk tests and visual inspection of Q–Q plots.

A significance level of α = 0.05 (5%) was used for hypothesis testing, consistent with standard practice in medical and biological research. Correlation and statistically significant differences were considered present when p < 0.05.

In addition to p-values, the magnitude of group differences was expressed using 95% confidence intervals for t-tests and ANOVA. Pearson’s correlation coefficient (r) was used to assess the strength of association between continuous variables (e.g., CD4^+^ cell counts and GMFI), with the corresponding p-value and coefficient of determination (R²) indicating the strength and fit of the linear relationship.

Frequency data were reported as percentages, allowing inference as to which group exhibited a higher incidence of the investigated condition.

## Results

### Antibody responses

Serum levels of anti-SARS-CoV-2 total IgG antibodies were measured on the day of vaccination, before vaccine administration, and 42–49 days after the first vaccination (21–28 days after the second vaccination), from which GMT and GMFI were calculated. Data are expressed as mean with 95% confidence interval (95%CI) (**Table 2**).

**Table 2:** Pre-vaccination GMT, post-vaccination GMT, and GMFI values in oncology patients and healthy control groups.

	Oncology patient (n=20)	Healthy controls (n=13)	P value
Pre-vaccination GMT (95%CI)	6.09 (3.55-10.43)	8.03 (3.34-19.30)	0.547
Post-vaccination GMT (95%CI)	40.47 (17.32-95.83)	334.66 (322.33-347.46)	**<0.001**
GMFI (95%CI)	6.69 (3.72-36.98)	41.67 (20.42-95.50)	**0.011**

GMT, geometric mean titer; GMFI, geometric mean fold increase; CI, confidence interval; n, number of participants; significant differences are bolded.

The post-vaccination GMT values were 40.47 vs. 334.66 (p < 0.001), and GMFI values were 6.69 vs. 41.67 (p = 0.011), revealing significant differences between the patient and control groups. Notably, the vaccine elicited a significantly higher antibody response in healthy participants than in patients.

There were no significant differences in pre- and post-vaccination seropositivity rates between the control group and patients. We measured seropositivity of 100% in healthy subjects and 75% in oncology patients after vaccination. Seroconversion was significantly higher (p = 0.023) in healthy controls (92.3%) compared to patients (55%) (**Table 3**).

**Table 3:** Relative frequency (percentage) of pre-vaccination seropositivity, post-vaccination seropositivity, and seroconversion in healthy and oncology patient groups.

	Oncology patient (n=20)	Healthy controls (n=13)	P value
Pre-vaccination seropositivity n (%)	6/20 (30.0)	5/13 (38.5)	0.714
Post-vaccination seropositivity n (%)	15/20 (75.0)	13/13 (100)	0.131
Seroconversion n (%)	11/20 (55.0)	12/13 (92.3)	**0.023**

n, number of participants; significant differences are bolded.

### Antibody responses in subgroups of oncology patients

We compared antibody responses among oncology patients by age group, tumor type, and treatment type (**Supplementary Table 2**). There were no significant differences in post-vaccination GMT and GMFI between any of the subgroups analyzed. The seroconversion rates were 66.7% vs. 45.5% in the 5–11 and 12–18 years groups; 60% vs. 50% in the solid tumor and hematology groups; and 72.7% vs. 33.3% in children on maintenance treatment compared to those on intensive treatment.

### The relationship between pre-vaccination lymphocyte subpopulations and the humoral immune response

As expected, in most cell populations, there is a significant difference in absolute cell counts and percentages analyzed by flow cytometry before vaccination between oncology patients and healthy controls (**Supplementary Table 3**).

**Supplementary Table 4** shows the absolute cell numbers and percentages analyzed using flow cytometry in oncology patients. By specifying the lymphocyte subpopulation counts, we examined the relations between these cells and the outcome of vaccination, based on GMT, GMFI (**Table 4**), and seroconversion (**Table 5**).

**Table 4:** The relations between different pre-vaccination cellular parameters and post-vaccination GMT and GMFI in oncology patients.

Baseline cellular parameters		P value post-vaccination GMT	P value GMFI
Total lymphocyte count	n/µl	**0.004 (r:0.617)**	**0.006 (r:0.588)**
CD3+	n/µl	**0.034 (r:0.517)**	**0.019 (r:0.562)**
CD4+	n/µl	**0.011 (r:0.601)**	**0.007 (r:0.629)**
CD4+ within lymphocytes	%	**0.022 (r:0.551)**	**0.021 (r:0.554)**
CD4+ within CD3 lymphocytes	%	**<0.001 (r:0.767)**	**0.005 (r:0.643)**
CD8+	n/µl	0.236	0.135
CD8+ within lymphocytes	%	0.478	0.643
CD8+ within CD3 lymphocytes	%	0.005* (r:0.650)	0.050* (r:0.482)
CD4/CD8 ratio		**<0.001 (r:0.750)**	**0.029 (r:0.529)**
CD3+CD56+	n/µl	0.651	0.349
CD4+CD3+CD56+	n/µl	**0.036 (r:0.512)**	**0.019 (r:0.560)**
CD8+CD3+CD56+	n/µl	0.646	0.992
DP CD3+CD56+	n/µl	0.166	0.084
DN CD3+CD56+	n/µl	0.715	0.853
iNKT	n/µl	0.868	0.923
CD3+CD56+ iNKT	n/µl	0.454	0.563
MAIT	n/µl	0.663	0.810
CD3+CD56+ MAIT	n/µl	0.226	0.388
γδ	n/µl	0.579	0.442
CD3+CD56+ γδ	n/µl	0.893	0.998
CD4+ iNKT	n/µl	0.788	0.655
CD4+ MAIT	n/µl	**0.013 (r:0.589)**	**0.010 (r:0.604)**
CD4+ MAIT within lymphocytes	%	**0.027 (r:0.534)**	**0.035 (r:0.514)**
CD4+ γδ	n/µl	0.786	0.971
CD8+ MAIT	n/µl	0.497	0.643
CD56+ NK	n/µl	0.237	0.217
CD56hi+ NK	n/µl	0.231	0.286
CD56low+ NK	n/µl	0.249	0.228
Other CD3+CD56+	n/µl	0.373	0.207
Other CD3+CD56+CD4+	n/µl	0.061	**0.039 (r:0.504)**
Other CD3+CD56+CD8+	n/µl	0.823	0.938
Other CD3+CD56+ DP	n/µl	0.216	0.091
Other CD3+CD56+ DN	n/µl	0.179	0.076

n, number; µl, microliter; GMT, geometric mean titer; GMFI, geometric mean fold increase; r, Pearson’s correlation coefficient; DP, double positive (CD4+CD8+) cells; DN, double negative (CD4–CD8–) cells; iNKT, invariant natural killer cells; MAIT, mucosal-associated invariant T cells; γδ, gamma-delta T cells; NK, natural killer cells; Other, other than iNKT, MAIT and γδ.

*negative correlation; significant differences are bolded.

**Table 5:** The relations between different pre-vaccination cellular parameters and post-vaccination seroconversion in oncology patients.

Baseline cellular parameters		Seroconverted n=8 mean ± SD	Not seroconverted n=9 mean ± SD	p value
Total lymphocyte count	n/µl	1378.18 ± 742.76	915.56 ± 411.59	0.097
CD3+	n/µl	639.13 ± 428.32	269.62 ± 209.13	0.051
CD4+	n/µl	378.58 ± 321.58	120.33 ± 121.04	0.062
CD4+ within lymphocytes	%	27.19 ± 12.71	12.72 ± 10.95	**0.023**
CD4+ within CD3 lymphocytes	%	58.20 ± 14.08	47.30 ± 14.41	0.136
CD8+	n/µl	175.05 ± 98.37	92.09 ± 62.56	0.053
CD8+ within lymphocytes	%	14.35 ± 6.53	11.16 ± 7.88	0.383
CD8+ within CD3 lymphocytes	%	32.92 ± 13.74	40.17 ± 13.94	0.299
CD4/CD8 ratio		2.16 ± 1.18	1.51 ± 1.15	0.266
CD3+CD56+	n/µl	35.33 ± 18.10	17.28 ± 15.83	**0.044**
CD4+CD3+CD56+	n/µl	9.83 ± 10.51	1.25 ± 1.30	0.055
CD8+CD3+CD56+	n/µl	17.86 ± 15.19	10.30 ± 12.12	0.272
DP CD3+CD56+	n/µl	0.78 ± 0.69	0.22 ± 0.18	0.055
DN CD3+CD56+	n/µl	6.88 ± 5.87	5.50 ± 6.09	0.642
iNKT	n/µl	1.29 ± 2.39	0.96 ± 1.48	0.731
CD3+CD56+ iNKT	n/µl	0.17 ± 0.32	0.18 ± 0.32	0.937
MAIT	n/µl	23.35 ± 19.83	20.07 ± 25.97	0.776
CD3+CD56+ MAIT	n/µl	3.86 ± 3.58	4.57 ± 5.68	0.765
γδ	n/µl	46.61 ± 53.59	33.25 ± 38.67	0.561
CD3+CD56+ γδ	n/µl	5.88 ± 4.07	4.99 ± 5.12	0.703
CD4+ iNKT	n/µl	0.47 ± 0.83	0.33 ± 0.39	0.652
CD4+ MAIT	n/µl	4.73 ± 3.37	1.84 ± 2.16	*0.050*
CD4+ MAIT within lymphocytes	%	36.38 ± 18.70	18.11 ± 15.25	**0.042**
CD4+ γδ	n/µl	10.55 ± 16.79	5.52 ± 12.96	0.497
CD8+ MAIT	n/µl	17.53 ± 15.98	16.57 ± 22.18	0.921
CD56+ NK	n/µl	118.79 ± 70.58	50.70 ± 52.54	**0.038**
CD56hi+ NK	n/µl	11.53 ± 8.53	6.10 ± 5.09	0.127
CD56low+ NK	n/µl	107.12 ± 64.04	44.57 ± 48.31	**0.037**
Other CD3+CD56+	n/µl	25.71 ± 18.97	6.69 ± 10.85	**0.030**
Other CD3+CD56+CD4+	n/µl	9.63 ± 11.78	0.93 ± 1.47	0.076
Other CD3+CD56+CD8+	n/µl	13.59 ± 15.30	5.05 ± 10.73	0.198
Other CD3+CD56+ DP	n/µl	0.50 ± 0.45	0.07 ± 0.06	**0.031**
Other CD3+CD56+ DN	n/µl	1.99 ± 2.07	0.64 ± 0.66	0.114

n, number; µl, microliter; SD, standard deviation; DP, double positive (CD4+CD8+) cells; DN, double negative (CD4–CD8–) cells; iNKT, invariant natural killer cells; MAIT, mucosal-associated invariant T cells; γδ, gamma-delta T cells; NK, natural killer cells; Other, other than iNKT, MAIT and γδ; significant differences are bolded.

**Figure 2:**
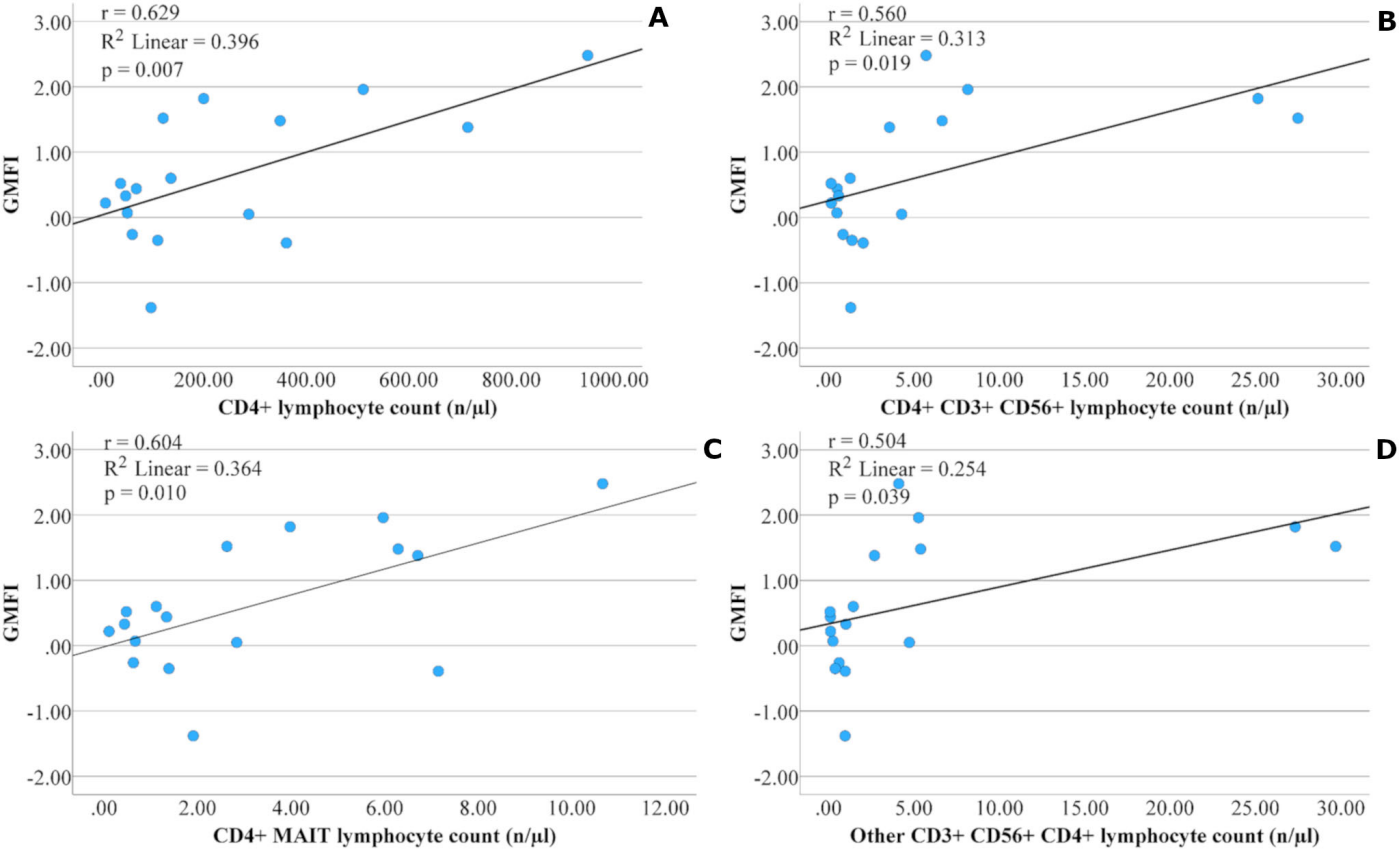
Relationship between the pre-vaccination number of special lymphocyte subsets and the antibody response (geometric mean fold increase, GMFI) in the oncology patient group after two doses of BNT162b2 mRNA COVID-19 vaccine. Subsets include: CD4^+^ T lymphocytes **(A)**; CD4^+^CD3^+^CD56^+^ T lymphocytes **(B)**; CD4^+^ MAIT cells **(C)**; and CD4^+^CD3^+^CD56^+^ T lymphocytes excluding iNKT, MAIT, and γδ T cells **(D)**. Pearson’s correlation coefficient (r), corresponding p-value, and coefficient of determination (R²) indicate the strength and fit of the linear relationship.

Our statistical analysis identified an association between the post-vaccination GMT, GMFI, and baseline absolute lymphocyte count (GMT: p = 0.004, GMFI: p = 0.006), CD3+ lymphocyte count (GMT: p = 0.034, GMFI: p = 0.019), and absolute CD4+ lymphocyte count (GMT: p = 0.011, GMFI: p = 0.007) (**Figure 2A**). We found the same association with the higher proportion of CD4+ lymphocytes within total lymphocytes (GMT: p = 0.022, GMFI: p = 0.021) and CD3+ lymphocytes (GMT: p<0.001, GMFI: p = 0.005). Conversely, a higher proportion of CD8+ lymphocytes within CD3+ cells showed a significant inverse relation (GMT: p = 0.005, GMFI: p = 0.050). In connection with this, a higher CD4/CD8 ratio was strongly associated with higher post-vaccination GMT (p<0.001) and GMFI (p = 0.029) values.

**Figure 3:**
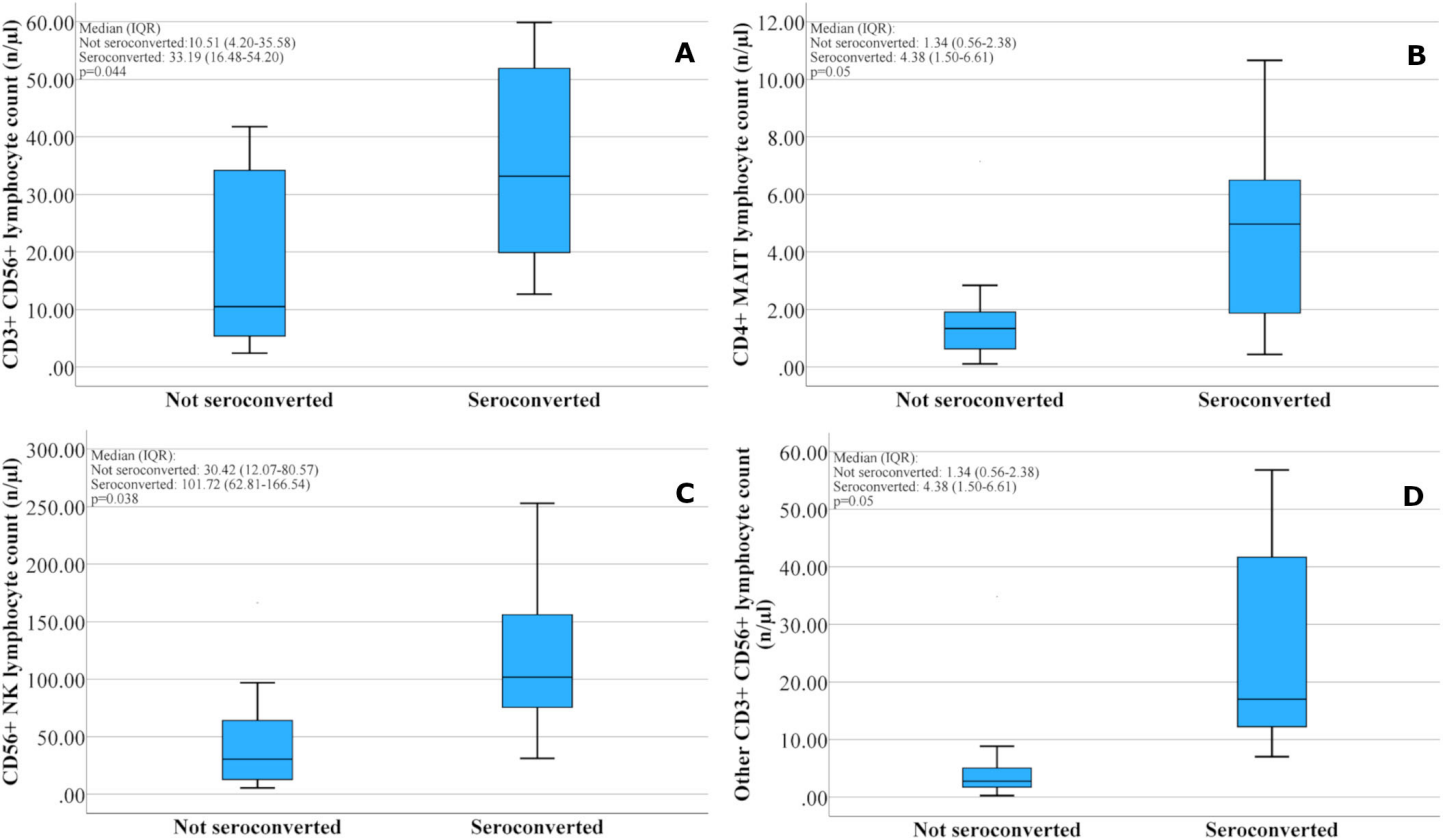
Relationship between the pre-vaccination number of special lymphocyte subsets and the seroconversion in the oncology patient group after two doses of BNT162b2 mRNA COVID-19 vaccine. Subsets include: CD3^+^CD56^+^T lymphocytes **(A)**; CD4^+^ MAIT cells **(B)**; CD56^+^ NK cells **(C)**; and CD3^+^CD56^+^ T lymphocytes excluding iNKT, MAIT, and γδ T cells **(D)**. Lymphocyte counts are presented as median values with interquartile ranges (IQRs) - number of patients: seroconverted n=8, not seroconverted n=9.

In patients with higher pre-vaccination CD3+CD56+ T cell counts (defined as [n/µl, mean ± SD] for each cell type), we observed a higher seroconversion rate (seroconverted: 35.33 ± 18.10; not seroconverted: 17.28 ± 15.83; p = 0.044) (**Figure 3A**). No correlation was found within CD3+CD56+ T cells for iNKT, MAIT, γδ T cells; however, the other (non-iNKT, MAIT, γδ T cells) CD3+CD56+ T cell population showed a relation with seroconversion (seroconverted: 25.71 ± 18.97, not seroconverted: 6.69 ± 10.85, p = 0.030) (**Figure 3D**). The characterization of this lymphocyte subpopulation is described in the gating strategy in the **Supplementary Figure 1** legend. Other (non-iNKT, MAIT, γδ T cells) CD3+CD56+ T cells were characterized as a set of CD56+ T cells that do not express Vα7.2, γδTCR, or Vα24–Jα18 TCR.

The presence of the CD4+ marker was also associated with a higher antibody response to vaccination in several other cell types. We also observed significant relations with seroconversion for percentage of CD4+ MAIT cells in lymphocytes (seroconverted: 36.38 ± 18.70, not seroconverted: 18.11 ± 15.25, p = 0.042), the other (non-iNKT, non-MAIT, non-γδ T cells) CD3+CD56+ DP cells (seroconverted: 0.50 ± 0.45, not seroconverted: 0.07 ± 0.06, p = 0.031), and with GMFI for CD4+ MAIT cells (p = 0.010) (**Figure 2C**), percentage of CD4+ MAIT cells in lymphocytes (p = 0.035), CD4+CD3+CD56+ cells (p = 0.019) (**Figure 2B**), and other (non-iNKT, non-MAIT, non-γδ T cells) CD3+CD56+CD4+ cells (p = 0.039) (**Figure 2D**). A tendency to significance was observed between absolute CD4+ MAIT cells and seroconversion (seroconverted: 4.73 ± 3.37, not seroconverted: 1.84 ± 2.16, p = 0.050) (**Figure 3B**).

It should be noted that these subsets are a heterogeneous population of unconventional and activated T cells, not phenotypically or functionally defined. Our findings with that small cohort of patients do not indicate any specific functional role of these cells. Therefore, distinguishing from our primary hypotheses (total lymphocytes, CD4+ T cells, CD4/CD8 ratio, CD3+CD56+ T cells, CD4+ MAIT cells), these should be considered exploratory analyses. However, they may have the potential to generate hypotheses.

Remarkably, a significant relationship was observed for both GMT, GMFI, and seroconversion in relation to the percentage of CD4+ MAIT cells and CD4+ MAIT cells within lymphocytes.

Non-parametric analyses (Spearman) confirmed the main associations (total lymphocytes, CD4+ T cells, CD3+CD56+ T cells, CD4+ MAIT cells), which remained statistically significant or closer to a statistically significant level.

In addition, CD56+ NK cells (seroconverted: 118.79 ± 70.58, not seroconverted: 50.70 ± 52.54, p = 0.038) (**Figures 3C**) and CD56low+ NK cells (seroconverted: 107.12 ± 64.04, not seroconverted: 44.57 ± 48.31, p = 0.037) showed a significant association with seroconversion.

No relation with the immune response was observed in the analysis of iNKT, MAIT, γδ T cells, and other cell populations carrying the CD8+ marker.

We also investigated the relationships between the pre-vaccination lymphocyte subpopulation and GMT, GMFI in healthy controls (we could not perform statistical analysis on seroconversion because all subjects were seropositive after vaccination). Only baseline CD56hi+ NK cell counts among healthy controls were significantly associated with post-vaccination GMT (p = 0.013).

### Flow cytometry results according to age-related reference values

We examined the humoral immune response to vaccination depending on whether specific pre-vaccination lymphocyte subpopulations were within the age-specific normal range (30, 31). It is important to note that age-specific reference ranges were not available for some lymphocyte subpopulations. In most oncology patients, the total lymphocyte count (55%), CD3+ (88.2%), CD4+ (82.4%), and CD8+ counts (70.6%) in significant proportions were below the lower limit of the age-specific intervals, and CD3+CD56+T cell counts (52.9%) were below the age-specific predicted values (30). In 70.6% of our patients, the CD4/CD8 ratio was within the normal range (**Supplementary Table 5**).

The antibody response to vaccination was significantly greater in those with pre-vaccination total lymphocyte count (post-vaccination GMT:107.15 vs. 18.45 (p = 0.028), GMFI: 21.83 vs. 2.55 (p = 0.049)), CD3+ (post-vaccination GMT: 342.77 vs. 21.02 (p<0.001), GMFI: 85.11 vs. 2.77 (p = 0.047)), CD4+ lymphocyte count (post-vaccination GMT: 252.93 vs. 18.37 (p = 0.015), GMFI: 87.10 vs. 2.15 (p = 0.008)), and CD4/CD8 ratio (post-vaccination GMT: 54.20 vs. 6.61 (p = 0.013), GMFI: 8.65 vs. 0.71 (p = 0.040)) were within the age-specific normal range (**Table 6**).

**Table 6:** The relations between the pre-vaccination values of different lymphocyte populations and post-vaccination GMT/GMFI/seroconversion in the oncology patient group, according to the value being in the age-specific normal range.

Pre-vaccination cellular parameters	Group (n)	Pre-vaccination GMT (95%CI)	P value	Post-vaccination GMT (95%CI)	P value	GMFI (95%CI)	P value	Seroconversion n (%)	P value
Total lymphocyte count	<norm (11)	7.28 (4.34-12.20)	0.491	18.45 (5.68-59.93)	**0.028**	2.55 (0.56-12.58)	**0.049**	5/11 (45.5%)	0.406
	≥norm (9)	4.90 (1.57-12.73)		107.15 (36.14-337.51)		21.83 (4.02-73.24)		6/9 (66.7%)	
CD3+	<norm (15)	7.60 (4.08-14.14)	0.481	21.02 (8.62-51.28)	**<0.001**	2.77 (0.86-8.93)	**0.047**	6/15 (40.0%)	0.206
	≥norm (2)	3.98 (0.02-1022.43)		342.77 (295.46-397.65)		85.11 (0.37-19789.37)		2/2 (100.0%)	
CD4+	<norm (14)	8.53 (4.61-15.80)	0.151	18.37 (7.38-45.72)	**0.015**	2.15 (0.70-6.65)	**0.008**	5/14 (35.7%)	0.082
	≥norm (3)	2.88 (0.22-38.09)		252.93 (95.99-666.43)		87.10 (8.50-892.38)		3/3 (100.0%)	
CD8+	<norm (12)	8.77 (4.54-16.95)	0.245	24.43 (8.39-71.15)	0.537	2.79 (0.95-8.21)	0.451	4/12 (33.3%)	0.131
	≥norm (5)	4.17 (0.83-20.89)		44.87 (4.08-493.79)		10.67 (0.19-586.82)		4/5 (80%)	
CD4/CD8 ratio	<norm (5)	9.33 (3.20-27.19)	0.463	6.61(1.71-25.49)	**0.013**	0.71(0.10-5.21)	**0.040**	1/5 (20%)	0.294
	≥norm (12)	6.28 (2.82-14.01)		54.20 (19.41-151.33)		8.65 (2.17-34.49)		7/12 (58.3%)	
CD3+CD56+**	<pred (9)	9.43 (3.56-24.97)	0.289	27.40 (8.11-92.62)	0.882	2.91 (0.77-11.03)	0.528	3/9 (33.3%)	0.347
	≥pred (8)	5.07 (2.26-11.38)		31.35 (5.87-167.41)		85.11 (0.56-66.93)		5/8 (62.5%)	

GMT, geometric mean titer; GMFI, geometric mean fold increase; CI, confidence interval; n, number of patients; **age-specific predicted values; norm: refers to the lower limit of age-related reference intervals; pred: refers to the age-specific predicted values of the CD3+CD56+ T cells; significant differences are bolded.

For several lymphocyte subpopulations, data on age-specific normal ranges are not available (**Supplementary Table 5**). One of the aims of the present study was to collect age-specific data on the proportions and absolute cell counts of each lymphocyte subpopulation in healthy children. However, due to drop-outs from the study, we were unable to perform this data collection.

### Adverse events

Local (local muscle pain or swelling) and systemic (generalized muscle pain, fatigue, fever) adverse reactions were reported after the first and second vaccinations. Among oncology patients, local reactions occurred in 13/20 cases (65%), generalized muscle pain in 1/20 cases (5%), and fatigue in 3/20 cases (15%) after the first vaccination; fever did not occur. After the second vaccination, local reactions occurred in 10/20 cases (50%), generalized muscle pain in 2/20 cases (10%), fatigue in 6/20 cases (30%), and fever in 3/20 cases (15%). No serious side effects were reported.

## Discussion

In this multicenter study, we investigated the immune responses at 3–4 weeks after two doses of BNT162b2 mRNA COVID-19 vaccine and the relationship between lymphocyte subpopulations measured before vaccination in Hungarian pediatric patients with solid or hematologic cancer and healthy children.

In our previous study (16), we demonstrated the predictive value of baseline absolute lymphocyte counts above 1000/µl. We found a relation between pre-vaccination CD3+CD56+ T cell counts and the antibody response against the trivalent influenza vaccine. The effect of the pre-vaccination CD4+ lymphocyte level within the normal age-specific range was observed on the serological response in children undergoing chemotherapy. In the present study, we sought evidence for the role of these factors in the immune response to COVID-19 mRNA vaccines.

Children with malignant disease have weakened immune systems as a consequence of the chemotherapy. This immunosuppressed state may persist for 6–12 months after the end of therapy (32).

A reduced lymphocyte count can significantly weaken humoral and cellular immunity, affecting the humoral defenses that may be present after previous vaccinations or infections. In addition, children’s immune response to vaccines may be inadequate during therapy and for months after anticancer treatment. In pediatric oncology patients, the antibody response rate to various viral and bacterial vaccines is variable (30%-88%) depending on the phase of therapy and the vaccine (33).

However, even if the response is limited, vaccination can prevent infection and reduce the severity of infection and mortality. Therefore, several guidelines and consensus statements provide recommendations for the vaccination of pediatric hematology and oncology patients (33, 34).

In our study, all patients received the vaccine at least 17–19 days after the start of the preceding chemotherapy block. No patient received the vaccine earlier, as the hematological nadir typically occurs 7–10 days after initiation of the chemotherapy cycle. According to the methodological guidelines of the Hungarian National Center for Epidemiology, vaccination was recommended during weeks 3–4 following the treatment cycle, when absolute neutrophil counts exceeded 0.5 G/L. We emphasize the importance of vaccination timing relative to chemotherapy. The standardized scheduling of our study strengthens internal validity but may limit generalizability in circumstances where different vaccination-chemotherapy intervals are used.

The first reviews published in connection with the coronavirus pandemic found that children with cancer are more likely to develop severe symptoms than the general child population and have a higher chance of complications and mortality. In a systematic review by Meena et al., the need for intensive care unit treatment was found to be 10.3%, and mortality attributable to COVID-19 was found to be 4.9% (4). These findings justified using the SARS-CoV-2 vaccine in pediatric oncology patients as soon as possible.

### Serological response

Regarding SARS-CoV-2 messenger RNA (mRNA) vaccines, Gilbert et al. found that COVID-19 vaccine efficacy correlates with the level of antibodies against the viral spike protein in adults (35).

Several studies have demonstrated that COVID-19 vaccination can induce an effective antibody response in oncology patients, even during treatment. Numerous reviews and meta-analyses discuss the efficacy and safety data for the adult cancer patient population. In these studies, the seroconversion rate after two doses of the mRNA COVID-19 vaccine in patients with cancer is varied, ranging between 47 and 95% (6–8, 36, 37), depending on the type of cancer and stage of treatment. Factors related to the patient population (advanced age, hematologic cancer) or treatment (intensive therapy or therapies associated with B-cell depletion) can lead to less robust immune responses following vaccination.

Due to differences in immune response, cancer types, treatment, and the much lower incidence of comorbidities, results from adult populations are hard to interpret when we aim to study immunological mechanisms in a pediatric setting, especially in children under 12 years of age.

Only a few studies analyzed the efficacy of the COVID-19 vaccine in pediatric cancer patients, involving a limited number of participants, and no study with a larger number of patients or a review had been published at the time of our study. Most of these (10, 38–40) included adolescents (over 12 years of age) and young adults, but data on patients under 12 years of age were explicitly scarce (41–45).

The corresponding antibody responses varied from 33% to 76.2%, depending on the therapy and tumor type. Typically, hematological patients and active treatment were associated with poorer immune response.

Our results are consistent with the findings of those studies and demonstrate similar antibody response rates to the vaccine.

Ma et al. analyzed data from 111 children with solid tumors (20 of whom were on active treatment) after administration of 2 doses of inactivated SARS-CoV-2 vaccine (CoronaVac), and found a 68.4% seropositivity rate (44).

Schmidt et al., following a 2- or 3-dose vaccination with BNT162b2 mRNA COVID-19 vaccine, found a good antibody response in 39.3% of patients (n = 28) who had been treated for less than 6 weeks and 94.4% of those (n = 18) who had been treated for more than 6 weeks with chemo/immunotherapy (for solid tumors and hematological malignancies) before the first immunization event (42).

Mastronuzzi et al. investigated the response to the BNT162b2 mRNA COVID-19 vaccine in oncology patients (n = 11 solid tumors, n = 14 hematological malignancies) aged 5–12 years with a seroconversion rate of 72.7% (solid tumors) and 66.7% (hematological malignancies) (45).

Previous studies have not addressed the effects of pre-vaccination immunosensitization and pre-vaccination seropositivity on antibody responses in COVID-19-vaccinated oncology patients. In some of our pre-vaccination seropositive patients, antibody titers decreased after repeated vaccination, suggesting that baseline serostatus may influence the intensity of the serological response. The reduced antibody response observed in previously seropositive patients following revaccination may be due to lymphocyte exhaustion.

### Relationship between pre-vaccination T-lymphocyte subpopulations and the humoral immune response

#### Relationship between baseline total lymphocyte count, CD3+, CD4+, CD8+ cell count, CD4/CD8 ratio and serological response

The data on the relationship between cellular immunity and the response to vaccines in children receiving chemotherapy for different malignancies are usually based on influenza vaccination studies. Positive correlations between baseline higher lymphocyte, CD3+, CD4+, and CD19+ counts and serological response have been described (12–14, 16, 17); conflicting results for CD8+ cells have been reported (13, 14).

About COVID-19 vaccination, Benda et al. described lower baseline lymphocyte count as a negative predictive marker of antibody response in adult oncology patients (18).

Our study observed significant correlations between total lymphocyte count, the absolute value of CD3+ and CD3+CD4+ lymphocyte count, a higher percentage of CD4+ cells among T lymphocytes, and the extent of antibody response. Similar to those for influenza vaccines (12, 13, 15–17), these findings have important implications. Notably, there was no correlation with the absolute number of CD8+ cells. A higher proportion of CD8+ cells within T lymphocytes showed an inverse correlation with postvaccination GMTs and GMFI. In accordance with these, the CD4/CD8 ratio is also positively associated with postvaccination GMT and GMFI.

The predictive value of the CD4/CD8 ratio for antibody response to the COVID-19 vaccine has only been investigated in HIV-positive patients (46, 47). A higher CD4/CD8 ratio has been found to be a positive predictor, but their data are inconsistent for CD4+ cells.

Similar results were obtained when the association between antibody response and total lymphocyte count, CD3+, CD4+, CD8+ lymphocyte count, and CD4/CD8 ratio was examined, depending on whether the value was within or below the age-specific reference range (30, 31). Total lymphocyte, CD3+, CD4+ cell counts, and CD4/CD8 ratios within the normal age-specific range are related to better immune response and significantly better results in post-vaccination GMT increase and GMFI. There was no such relation with CD8+ cells.

Our results suggest that among the cellular components of the adaptive immune system, CD3+CD4+ cells play a prominent role in the humoral immune response to the BNT162b2 mRNA COVID-19 vaccine, even in immunosuppressed children.

#### Relationship between baseline NK cell count and serological response

Our results indicated that baseline CD56+ NK cell counts were related to significantly higher seroconversion rates in the pediatric oncology population.

NK cells are innate lymphoid cells. During infection, they are among the first to respond to pathogens and contribute to the early clearance of viral infections (48). However, NK cells can also enhance and influence the subsequent adaptive response through the secretion of cytokines and chemokines (49, 50). The main cytokines are γ-interferon (IFN-γ), tumor necrosis factor-α, granulocyte-macrophage colony-stimulating factor, IL-10, and IL-13 (51).

NK cells can regulate adaptive immunity positively and negatively, and the mechanism of immunomodulation is context-dependent (52). Activating antigen-presenting cells by vaccines or adjuvants increases and sustains NK cell activity, contributing to T cell recruitment and memory cell formation (53). Consistent with our results, Cuapio et al. found a positive correlation between NKG2C+ NK cell subsets and anti-SARS-CoV-2 antibody titers (54).

Benda et al. found that a lower baseline natural killer cell count was a negative predictive marker of antibody response to the COVID-19 vaccine in adult oncology patients (18).

#### Relationship between baseline CD3+CD56+ T cells, invariant natural killer T cells, gamma delta T cells, mucosal-associated invariant T cells, and serological response

We have previously found a positive association between baseline CD3+CD56+ T cell count and post-vaccination antibody response following influenza vaccination among pediatric oncology patients (16). Therefore, we investigated the relation of pre-vaccination CD3+CD56+ T cell subpopulations and unconventional T cells (iNKT, MAIT, γδ T cells) with the antibody response to the COVID-19 vaccine in oncology patients undergoing treatment.

We observed that higher pre-vaccination CD3+CD56+ cell counts were related to a higher seroconversion rates. The possible positive effect of CD4+ CD3+CD56+ T cells on post-vaccination GMT and GMFI was even more pronounced. However, no relation was found based on whether the baseline CD3+CD56+ T cell count was above the age-specific predicted value. No relation was found for total iNKT, MAIT, and γδ T cells, nor for CD3+CD56+ iNKT, MAIT, and γδ T cells. In addition, the association between the other CD3+CD56+ T cell group (other than iNKT, MAIT, and γδ T cells) and seroconversion was observed to be significant. The CD4+ marker-carrying sub-group and the double positive (CD4+CD8+) sub-group within this cell group may contribute to a more potent serological response (GMFI and seroconversion).

As mentioned above, these data should be evaluated with caution and considered as exploratory analyses, emphasizing their hypothesis-generating potential.

From animal data (55, 56), we know that iNKT (CD1d-restricted) cells are responsible for a significant part of the production of B-cell modulating cytokines (IL-4, IFN-γ) in the early stages of viral infections.

Several studies have reported that circulating CD3+CD56+ T cell frequencies are a predictive biomarker for severe COVID-19 disease (23, 24). In some clinical analyses, CD3+CD56+ T cells have been defined as NKT-like cells (CD3+CD56+ T cells with an NKT-like phenotype) (57, 58). The activation of unconventional or innate-like T cells (such as γδ T cells, iNKT cells, and MAIT cells) has also been associated with the course of COVID-19 (19, 59). These cells display features of innate and adaptive immune systems, currently mentioned as cells of the natural immune system, most of which do not express CD56 (60–62).

CD3+CD56+ T cells are a highly diverse group of cells comprising many subpopulations. Romero-Olmedo et al. have described 19 phenotypically distinct CD3+CD56+ T cells, classifying them into five major clusters (CD56+ population among TCRγδ+ T cells; CD4+ cells; CD8+ cells; iNKT cells (CD4– CD8– T cells expressing diverse or invariant (CD1d-restricted) αβ chains of the TCR); and MAIT cells (TCRVα7.2+CD161+ mucosal-associated invariant T cells) (63).

According to the studies mentioned, the heterogeneous population of the CD3+CD56+ cells and unconventional T cells may contribute to antiviral immunity in several ways. Our data obtained from the small cohort may support this hypothesis. However, other explanations are also possible, e.g., that these subsets may simply mark overall T-cell robustness rather than being direct effectors of the antibody response, or that their presence may indicate the more rapid repopulation of innate-like cells during the post-chemotherapy immune reconstitution process.

Another important finding is that the pre-vaccination CD4+ MAIT cell count may contribute to a more significant antibody response rate based on post-vaccination GMT, GMFI, and seroconversion. Mucosal-associated invariant T (MAIT) cells are unconventional T cells with antimicrobial responsiveness characteristic of the innate immune system. MAIT cells typically recognize microbial riboflavin metabolites (including the potent agonist 5-(2-oxopropylidene-amino)-6-d-ribolylaminouracil (5-OP-RU)) MR1 (MHC I-class-related protein 1) in a restricted manner, allowing them to respond to a wide range of microbes (64, 65). MAIT cells can also respond to various viral infections in mouse models and also in humans (64, 66, 67). MAIT cells are activated early in a viral infection, supporting the protective host response by facilitating early cytokine production and the development of an adaptive immune response. SARS-CoV-2 infection triggers a strong activation of MAIT cells, which in severe infection causes a rapid reduction in the number of circulating MAIT cells due to their accumulation in the airways (19–21). Provine et al. demonstrated that a leading adenovirus vector vaccine, ChAdOx1 (Chimpanzee Adenovirus Oxford 1, also used as a backbone for SARS-CoV-2 vaccines), activated MAIT cells in immunized mice and human volunteers (68).

Jensen et al. described Tfh-like phenotypic MAIT cells in human lymphoid tissue that promoted B-cell differentiation and enhanced specific immunoglobulin responses (69). It was demonstrated using mouse models that these Tfh-like MAIT cells may play a role in modulating antibody responses to bacterial vaccines. Rahmann et al. reported that MAIT cell numbers correlated with Simian immunodeficiency virus (SIV) specific antibodies and SIV-specific memory B cells when rhesus macaques were vaccinated with an adenovirus-SIV vaccine (70). Among people living with HIV (PLWH) or with primary immunodeficiency (PID) and healthy donors (HD), Boulois et al. found that pre-vaccination MAIT cell count is associated with humoral and CD4 T cell responses following BNT162b2 mRNA vaccination, but the least in PLWH patients (22). MAIT cell activation is assumed to occur at the vaccination site or in regional lymph nodes, as no changes in the circulating MAIT cell population were observed after vaccination. These data support the idea that MAIT cells are essential participants in developing adaptive and humoral immune responses. It is hypothesized that CD4 positivity confers an additional helper function to MAIT cells. This, by altering the cytokine profile, positively influences the activation of adaptive immune cells and the adaptive immune response. Based on these findings, several authors have raised the possibility of using stimulatory MR1 ligands to boost MAIT cell frequencies to enhance innate viral defenses in vaccination (71–73).

The vaccine was safe and well-tolerated, even in pediatric populations with altered immune status, without severe adverse events, consistent with other studies in pediatric oncology patients (9, 38, 44). However, it is important to note that, in patients with altered immune systems, although SARS-CoV-2 mRNA vaccines are safe, after an additional booster dose, T-cell exhaustion might develop due to repeated priming with the same antigen (74). Vaccine-related adverse effects did not result in treatment delays. Most of the local and systemic adverse events we observed after vaccination were low-grade. As other studies reported (39, 75), the incidence of local adverse reactions decreased, while that of systemic adverse reactions increased after the second (and third) vaccination.

Our study has several limitations. The statistical analyses were limited by the small number of patients enrolled and the heterogeneous population. In addition, although a limited number of primary hypotheses were predefined, no formal adjustment for multiple testing was applied; therefore, the risk of type I error cannot be excluded, and the results—particularly the exploratory findings—should be interpreted with caution. The heterogeneity of diagnoses and treatments may be a potential confounding factor in interpreting the associations between baseline immune subsets and vaccine responses. Although an initial sample size calculation was conducted, the final number of vaccinated oncology patients was lower than expected due to the limited availability of eligible participants and decreased willingness to be vaccinated during the enrollment period. As a result, the statistical power for subgroup comparisons and correlation analyses was limited, and these findings should be regarded as exploratory and for generating hypotheses. Larger multicenter studies will be needed to verify these preliminary results. Five participants in the healthy control group refused a second blood draw; therefore, the control group data were limited.

The lack of neutralization assays is considered a limitation of the study. We did not investigate the cellular immune response following vaccination, an important factor in vaccine-induced immunity, and the relationship between different cell groups following vaccination and the humoral immune response. We did not measure SARS-CoV-2-specific T-cell function.

All these limitations caution against overinterpreting the number of phenotypic subgroups in terms of functional antiviral T-cell immunity.

Despite these limitations, the current results contribute to our knowledge of COVID-19 vaccination during and after cancer treatment, specifically in younger children.

## Conclusions

Our data suggest that humoral immune responses can be elicited by the SARS-CoV-2 vaccine in a significant proportion of pediatric patients receiving intensive or maintenance therapy for cancer. However, the magnitude of the response is lower than in healthy children. In pediatric oncology patients, we described for the first time the possible relationship between pre-vaccination T-lymphocyte subpopulations (CD4+, CD3+CD56+, CD4+CD3+CD56+, CD56+ NK cells) and the antibody response to the COVID-19 vaccine. Our observations with the SARS-CoV-2 vaccine are consistent with our previous hypothesis with the influenza vaccine, suggesting that CD3+CD56+ T cells may participate in the immune response to these vaccines, which may be related to the possible role of CD4+CD3+CD56+ T cells in this. In our small but highly immunosuppressed study population, we highlight possible connections between pre-vaccination CD4+ MAIT cell population and post-vaccination GMT, GMFI, and seroconversion, although these preliminary associations require validation.

We explicitly emphasize that larger, ideally multicenter studies are needed to confirm these preliminary findings, with standard vaccination timing and functional T-cell assays. It would be necessary to determine the age-specific normal ranges of lymphocyte groups in childhood in a larger population, and based on these, to investigate the role of these cells in the humoral and cellular immune response to vaccination in a larger cohort.

## Data Availability

The original contributions presented in the study are included in the article/**Supplementary Material**. Further inquiries can be directed to the corresponding author.
