# Supplementary material for: Possible role of pre-vaccination T-lymphocyte subpopulations in the antibody response to COVID-19 vaccines in children undergoing chemotherapy
Source: Front Immunol. 2026 Feb 5;17:1728845. doi: 10.3389/fimmu.2026.1728845 (PMC12916594; doi:10.3389/fimmu.2026.1728845)
Supplement: Supplementary file 1 [file Table1.doc]

Supplementary Material

# Supplementary Figures and Tables

## Supplementary Figures

**Supplementary Figure 1.** Flow cytometry strategy: First, CD4+ T helper and CD8+ cytotoxic T cells were identified within CD3+ T lymphocytes. Then, NK cells were gated as CD3-CD56+ cells (including CD56hi+ and CD56low+ populations); and the total CD3+CD56+ population was also gated in the same plot. MAIT cells were identified as Vα7.2+ T cells, γδT cells as γδTCR+ T cells, and iNKT cells as Vα24-Jα18+ T cells. The ratio of CD4+, CD8+, CD4 and CD8 double negative (DN), and double positive (DP) cells; as well as the ratio of CD56+ cells were measured within MAIT, γδT, and iNKT cells, respectively. We realized that CD56+ MAIT, γδT, and iNKT cells together are fewer than the total CD3+ CD56+ cells. Thus, we defined the remaining CD3+ CD56+ cells as “other” CD3+ CD56+ cells, gated with a Boolean strategy in the analysis software as follows: CD3+ CD56+ and non-MAIT, non-γδT, non-iNKT cells (the set of CD56+ T cells that do not express Vα7.2, γδTCR, Vα24–Jα18 TCR). Finally, the ratio of CD8+, CD4+, CD4 and CD8 double negative (DN) and double positive (DP) cells were also measured within total CD3+CD56+ cells, as well as its 4 subsets: CD56+ MAIT, CD56+ γδT, CD56+ iNKT, and “other” (non-MAIT, non-γδT, non-iNKT) CD3+ CD56+ cells, respectively.

## Supplementary tables

**Supplementary Table 1.** The data of the oncology patients in the study by sex, age, tumor, and treatment type.

| **Patients** | **Age (years)** | **Sex** | **Malignancy** | **Treatment** |
| --- | --- | --- | --- | --- |
| 1. | 17 | F | Hodgkin lymphoma | intensive |
| 2. | 12 | F | Osteosarcoma | intensive |
| 3. | 9 | F | CNS tumor (astrocytoma) | intensive |
| 4. | 16 | M | T-cell lymphoblastic lymphoma | maintenance |
| 5. | 5 | M | ALL | intensive |
| 6. | 10 | M | ALL | maintenance |
| 7. | 15 | F | Non-Hodgkin lymphoma | intensive |
| 8. | 14 | M | ALL | maintenance |
| 9. | 9 | M | ALL | maintenance |
| 10. | 8 | M | Hodgkin lymphoma | intensive |
| 11. | 6 | F | CNS tumor (astrocytoma) | maintenance |
| 12. | 17 | M | Rhabdomyosarcoma | intensive |
| 13. | 5 | M | ALL | maintenance |
| 14. | 5 | M | Rhabdomyosarcoma | intensive |
| 15. | 14 | F | ALL (Precursor T-cell) | maintenance |
| 16. | 14 | F | Osteosarcoma | intensive |
| 17. | 6 | F | ALL | maintenance |
| 18.* | 14 | M | Ewing sarcoma | maintenance |
| 19.* | 18 | F | Epitheloid sarcoma | maintenance |
| 20.* | 15 | M | Wilms tumor | maintenance |

*F: female; M: male; CNS: central nervous system; ALL: acute lymphoblastic leukemia; *: the data of these patients were evaluated only concerning the antibody response to vaccination.*

**Supplementary Table 2**. Pre-vaccination GMT, post-vaccination GMT, GMFI values, and relative frequency (percentage) of seroconversion in oncology patient groups based on age, the type of cancer, and therapy.

|  | **n** | **Pre-vaccination GMT** (95%CI) | **p value** | **Post-vaccination** **GMT** (95%CI) | **p value** | **GMFI** (95%CI) | **p value** | **Seroconversion** (%) | **p value** |
| --- | --- | --- | --- | --- | --- | --- | --- | --- | --- |
| **5-11 years** | 9 | 6.37  (2.62-15.46) | 0.877 | 75.51  (22.54-252.93) | 0.179 | 11.86  (8.59-16.36) | 0.365 | 6/9 (66.7) | 0.406 |
| **12-18 years** | 11 | 5.86  (2.61-13.14) |  | 24.60  (6.70-90.36) |  | 4.20  (2.56-6.88) |  | 5/11 (45.5) |  |
|  | | | | | | | | | |
| **Solid** | 10 | 4.55  (1.77-11.69) | 0.269 | 53.33  (13.40-212.32) | 0.524 | 11.72  (1.32-103.28) | 0.327 | 6/10 (60.0) | 1 |
| **Hematology** | 10 | 8.15  (4.07-16.22) |  | 31.12  (8.58-112.20) |  | 3.82  (1.10-13.27) |  | 5/10 (50.0) |  |
|  | | | | | | | | | |
| **Intensive treatment** | 9 | 9.79  (3.40-28.25) | 0.122 | 26.51  (7.46-93.97) | 0.355 | 2.71  (0.41-17.54) | 0.138 | 3/9 (33.3) | 0.175 |
| **Maintenance treatment** | 11 | 4.06  (2.38-6.92) |  | 73.28  (20.94-256.45) |  | 18.07  (4.13-79.80) |  | 8/11 (72.7) |  |

*GMT: geometric mean titer; GMFI: geometric mean fold increase; CI: confidence interval; n:number of patients*

**Supplementary Table 3**. Mean absolute number and percentage of T-lymphocytes in oncology patients and healthy controls before vaccination

| **Baseline cellular parameters** |  | **oncology patient**  n=17  mean ±SD | **healthy controls**  n=13  mean ±SD | **p value** |
| --- | --- | --- | --- | --- |
| Total lymphocyte count | n/µl | 1170.00±646.10 | 2137.69±785.45 | **<0.001** |
| CD3+ | n/µl | 413.46±368.54 | 1117.95±496.04 | **<0.001** |
| CD4+ | n/µl | 241.86±265.00 | 644.35±317.22 | **<0.001** |
| CD4+ within lymphocytes | % | 19.53±13.64 | 28.51±7.19 | **0.014** |
| CD4+ within CD3 lymphocytes | % | 52.43±14.90 | 56.38±6.31 | 0.336 |
| CD8+ | n/µl | 131.13±89.51 | 342.61±139.93 | **<0.001** |
| CD8+ within lymphocytes | % | 12.66±7.24 | 15.78±4.54 | 0.185 |
| CD8 within CD3 lymphocytes | % | 36.76±13.92 | 31.41±5.36 | 0.161 |
| CD4/CD8 ratio |  | 1.82±1.18 | 1.87±0.50 | 0.875 |
| CD3+CD56+ | n/µl | 25.77±18.84 | 68.26±99.88 | 0.155 |
| CD4+CD3+CD56+ | n/µl | 5.29±8.28 | 23.19±70.03 | 0.377 |
| CD8+CD3+CD56+ | n/µl | 13.86±13.76 | 26.83±27.96 | 0.143 |
| DP CD3+CD56+ | n/µl | 0.48±0.55 | 1.16±1.86 | 0.226 |
| DN CD3+CD56+ | n/µl | 6.15±5.84 | 17.13±14.52 | **0.021** |
| iNKT | n/µl | 1.11±1.91 | 5.04±5.59 | **0.029** |
| CD3+CD56+ iNKT | n/µl | 0.17±0.31 | 0.92±1.49 | 0.099 |
| MAIT | n/µl | 21.62±22.63 | 68.13±48.25 | **0.005** |
| CD3+CD56+ MAIT | n/µl | 4.24±4.68 | 11.43±12.66 | 0.071 |
| γδ | n/µl | 39.54±45.29 | 141.51±121.27 | **0.012** |
| CD3+CD56+ γδ | n/µl | 5.41±4.53 | 16.65±17.14 | **0.038** |
| CD4+ iNKT | n/µl | 0.39±0.62 | 2.54±2.92 | **0.022** |
| CD4+ MAIT | n/µl | 3.20±3.08 | 10.28±6.53 | **0.002** |
| CD4+ MAIT within lymphocytes | % | 26.71±18.91 | 44.85±23.39 | **0.026** |
| CD4+ γδ | n/µl | 7.89±14.63 | 30.92±54.16 | 0.159 |
| CD8+ MAIT | n/µl | 17.02±18.92 | 55.04±42.17 | **0.008** |
| CD56+ NK | n/µl | 82.74±69.19 | 220.47±149.86 | **0.002** |
| CD56+ hi NK | n/µl | 8.65±7.25 | 16.15±9.56 | **0.021** |
| CD56+ low NK | n/µl | 74.00±63.22 | 204.33±148.96 | **0.003** |
| Other CD3+CD56+ | n/µl | 15.64±17.66 | 33.69±84.55 | 0.463 |
| Other CD3+CD56+CD4+ | n/µl | 5.02±9.04 | 19.19±63.84 | 0.442 |
| Other CD3+CD56+CD8+ | n/µl | 9.07±13.39 | 11.77±19.18 | 0.652 |
| Other CD3+CD56+ DP | n/µl | 0.27±0.37 | 0.51±0.94 | 0.352 |
| Other CD3+CD56+ DN | n/µl | 1.28±1.61 | 2.20±5.08 | 0.480 |

*SD:standard deviation; DP: double positive (CD4+CD8+) cells; DN: double negative (CD4–CD8–) cells;* *iNKT: invariant natural killer cells; MAIT: mucosal-associated invariant T cells; γδ: gamma-delta T cells; NK: natural killer cells; n:number; µl: microliter; Other: other than iNKT, MAIT, γδ*

**Supplementary Table 4.** Pre-vaccination and post-vaccination anti-SARS-CoV-2 IgG levels, absolute number, and percentage of T lymphocytes in oncology patients before vaccination

| **Patients** | **1.** | **2.** | **3.** | **4.** | **5.** | **6.** | **7.** | **8.** | **9.** | **10.** | **11.** | **12.** | **13.** | **14.** | **15.** | **16.** | **17.** | **18.** | **19.** | **20.** |
| --- | --- | --- | --- | --- | --- | --- | --- | --- | --- | --- | --- | --- | --- | --- | --- | --- | --- | --- | --- | --- |
| **Pre-vaccination Anti-SARS-CoV-2 IgG (RU/ml)** | 2.3 | 38.5 | 2.9 | 7.2 | 3.6 | 4.9 | 20.3 | 7.4 | 4.1 | 61.6 | 1.1 | 35.7 | 6.4 | 13.2 | 2.8 | 1.5 | 14.6 | 3.3 | 4.5 | 1.3 |
| **Post-vaccination Anti-SARS-CoV-2 IgG (RU/ml)** | 6.4 | 15.6 | 88.4 | 4 | 119.5 | 5.5 | 9.1 | 29.2 | 330.9 | 204.6 | 328.5 | 1.5 | 13.6 | 15.5 | 4.6 | 138.1 | 353.4 | 167.4 | 311.5 | 383.2 |
| **Seroconversion** | 0 | 0 | 1 | 0 | 1 | 0 | 0 | 1 | 1 | 0 | 1 | 0 | 1 | 0 | 0 | 1 | 1 | 1 | 1 | 1 |
| **Total lymphocyte count (n/µl)** | 1030 | 1460 | 1850 | 570 | 710 | 810 | 770 | 800 | 560 | 1610 | 2600 | 870 | 400 | 840 | 280 | 1570 | 1490 | 1920 | 950 | 2310 |
| **CD3+ (n/µl)** | 164.29 | 574.22 | 706.89 | 172.14 | 313.89 | 529.25 | 259.26 | 405.52 | 315.78 | 56.67 | 1414.7 | 491.38 | 141.56 | 159.26 | 20.08 | 803.06 | 1011.7 |  |  |  |
| **CD4+ (n/µl)** | 68.48 | 361.30 | 349.07 | 60.63 | 120.83 | 287.93 | 110.29 | 135.95 | 199.85 | 37.84 | 949.03 | 97.20 | 47.64 | 50.97 | 8.31 | 511.11 | 715.18 |  |  |  |
| **CD4+ within lymphocytes (%)** | 6.65 | 24.75 | 18.87 | 10.64 | 17.02 | 35.55 | 14.32 | 16.99 | 35.69 | 2.35 | 36.50 | 11.17 | 11.91 | 6.07 | 2.97 | 32.56 | 48.00 |  |  |  |
| **CD4+ within CD3 lymphocytes (%)** | 50.18 | 68.55 | 54.33 | 32.84 | 45.07 | 54.04 | 45.50 | 35.41 | 73.34 | 66.38 | 67.57 | 24.48 | 50.15 | 42.40 | 41.34 | 66.29 | 73.46 |  |  |  |
| **CD8+ (n/µl)** | 54.55 | 125.91 | 225.78 | 114.87 | 113.40 | 165.49 | 109.25 | 233.08 | 50.98 | 9.71 | 312.22 | 180.71 | 32.32 | 58.65 | 9.64 | 216.81 | 215.84 |  |  |  |
| **CD8+ within lymphocytes (%)** | 5.30 | 8.62 | 12.20 | 20.15 | 15.97 | 20.43 | 14.19 | 29.13 | 9.10 | 0.60 | 12.01 | 20.77 | 8.08 | 6.98 | 3.44 | 13.81 | 14.49 |  |  |  |
| **CD8+ within CD3 lymphocytes (%)** | 39.97 | 23.89 | 35.14 | 62.22 | 42.30 | 31.06 | 45.07 | 60.71 | 18.71 | 17.04 | 22.23 | 45.51 | 34.02 | 48.79 | 47.94 | 28.12 | 22.17 |  |  |  |
| **CD4/CD8 ratio** | 1.26 | 2.87 | 1.55 | 0.53 | 1.07 | 1.74 | 1.01 | 0.58 | 3.92 | 3.90 | 3.04 | 0.54 | 1.47 | 0.87 | 0.86 | 2.36 | 3.31 |  |  |  |
| **CD3+ CD56+ (n/µl)** | 6.70 | 10.51 | 47.36 | 41.78 | 59.92 | 34.18 | 14.55 | 56.48 | 34.66 | 2.42 | 26.78 | 36.98 | 13.04 | 5.38 | 3.02 | 31.71 | 12.67 |  |  |  |
| **CD4+ CD3+ CD56+ (n/µl)** | 0.52 | 2.04 | 6.66 | 0.86 | 27.48 | 4.29 | 1.39 | 1.28 | 25.14 | 0.16 | 5.72 | 1.31 | 0.6 | 0.5 | 0.17 | 8.16 | 3.58 |  |  |  |
| **CD8+ CD3+ CD56+ (n/µl)** | 5.15 | 4.09 | 20.54 | 37.96 | 20.66 | 17.82 | 7.7 | 52.08 | 6.66 | 0.64 | 10.66 | 16.18 | 8.96 | 2.52 | 0.67 | 18.53 | 4.77 |  |  |  |
| **DP CD3+ CD56+ (n/µl)** | 0.21 | 0.44 | 0.93 | 0.17 | 2.06 | 0.24 | 0.39 | 0.16 | 0.39 | 0 | 0.26 | 0.44 | 0.44 | 0.08 | 0 | 1.57 | 0.45 |  |  |  |
| **DN CD3+ CD56+ (n/µl)** | 0.82 | 3.94 | 19.24 | 2.79 | 9.73 | 11.91 | 5.01 | 2.96 | 2.46 | 1.45 | 10.14 | 19.14 | 3.04 | 2.27 | 2.18 | 3.45 | 4.02 |  |  |  |
| **iNKT (n/µl)** | 1.34 | 1.61 | 1.67 | 0.17 | 0.14 | 0.08 | 0.15 | 0.32 | 0.17 | 0.16 | 0.52 | 4.61 | 0.12 | 0.17 | 0.31 | 7.07 | 0.30 |  |  |  |
| **CD3+ CD56+ iNKT (n/µl)** | 0.31 | 0.29 | 0.19 | 0.06 | 0.07 | 0 | 0 | 0.08 | 0.06 | 0 | 0 | 0.96 | 0 | 0 | 0 | 0.94 | 0 |  |  |  |
| **MAIT (n/µl)** | 16.07 | 18.98 | 58.65 | 6.90 | 16.33 | 34.34 | 14.94 | 9.28 | 6.27 | 1.61 | 35.36 | 83.26 | 1.16 | 4.20 | 0.36 | 40.98 | 18.77 |  |  |  |
| **CD3+ CD56+ MAIT(n/µl)** | 2.27 | 1.17 | 10.92 | 2.51 | 4.05 | 14.82 | 5.78 | 4.64 | 2.18 | 0.32 | 1.56 | 13.40 | 0.04 | 0.84 | 0.03 | 6.59 | 0.89 |  |  |  |
| **γδ (n/µl)** | 6.90 | 87.45 | 29.42 | 7.75 | 8.45 | 68.61 | 10.55 | 25.28 | 10.58 | 10.63 | 158.60 | 95.44 | 14.24 | 10.67 | 1.26 | 98.28 | 28.01 |  |  |  |
| **CD3+ CD56+ γδ (n/µl)** | 0.72 | 4.96 | 2.59 | 2.85 | 4.97 | 11.18 | 3.54 | 4.80 | 3.08 | 1.93 | 15.34 | 15.83 | 3.96 | 2.94 | 1.04 | 7.07 | 5.22 |  |  |  |
| **CD4+ iNKT (n/µl)** | 0.21 | 1.02 | 0.37 | 0.11 | 0.14 | 0 | 0.08 | 0.08 | 0.11 | 0.16 | 0.26 | 0.96 | 0.12 | 0.08 | 0.31 | 2.51 | 0.15 |  |  |  |
| **CD4+ MAIT (n/µl)** | 1.34 | 7.15 | 6.29 | 0.63 | 2.63 | 2.84 | 1.39 | 1.12 | 3.98 | 0.48 | 10.66 | 1.91 | 0.44 | 0.67 | 0.11 | 5.97 | 6.71 |  |  |  |
| **CD4+ MAIT within lymphocytes (%)** | 0.13 | 0.49 | 0.34 | 0.11 | 0.37 | 0.35 | 0.18 | 0.14 | 0.71 | 0.03 | 0.41 | 0.22 | 0.11 | 0.08 | 0.04 | 0.38 | 0.45 |  |  |  |
| **CD4+ γδ (n/µl)** | 0.21 | 40 | 3.15 | 0.51 | 2.91 | 3 | 1.69 | 3.44 | 3.02 | 0.64 | 15.6 | 1.91 | 0.68 | 1.51 | 0.2 | 50.55 | 5.07 |  |  |  |
| **CD8+ MAIT (n/µl)** | 14.11 | 11.39 | 45.7 | 6.21 | 14.2 | 29.08 | 12.94 | 7.44 | 2.8 | 0.97 | 23.92 | 70.82 | 0.72 | 3.36 | 0.25 | 34.7 | 10.73 |  |  |  |
| **CD56+ NK (n/µl)** | 20.09 | 166.29 | 252.71 | 97.01 | 100.68 | 48.84 | 30.42 | 177.04 | 50.18 | 12.72 | 101.66 | 64.12 | 31.24 | 11.42 | 5.35 | 135.02 | 101.77 |  |  |  |
| **CD56+ hi NK (n/µl)** | 5.87 | 17.52 | 27.94 | 4.73 | 16.4 | 8.91 | 2.31 | 9.44 | 2.24 | 1.93 | 9.62 | 7.83 | 2.52 | 5.29 | 0.48 | 7.54 | 16.54 |  |  |  |
| **CD56+ low NK (n/µl)** | 14.21 | 148.63 | 224.41 | 92.4 | 84.14 | 39.93 | 27.95 | 167.36 | 47.88 | 10.79 | 92.04 | 56.29 | 28.68 | 6.05 | 4.87 | 127.48 | 84.93 |  |  |  |
| **Other CD3+ CD56+ (n/µl)** | 2.74 | 2.70 | 18.32 | 34.83 | 56.83 | 8.83 | 3.03 | 50.54 | 32.82 | 0.25 | 9.48 | 5.03 | 14.95 | 1.09 | 1.72 | 15.71 | 7.01 |  |  |  |
| **Other CD3+ CD56+ CD4+(n/µl)** | 0.12 | 0.99 | 5.4 | 0.63 | 29.69 | 4.74 | 0.4 | 1.46 | 27.32 | 0.1 | 4.12 | 0.98 | 1.03 | 0.27 | 0.13 | 5.28 | 2.7 |  |  |  |
| **Other CD3+ CD56+ CD8+(n/µl)** | 2.51 | 1.13 | 8.85 | 33.51 | 19.63 | 1.85 | 1.69 | 48.78 | 4.29 | 0.05 | 2.9 | 3.62 | 12.47 | 0.52 | 0.53 | 9.19 | 2.59 |  |  |  |
| **Other CD3+ CD56+ DP (n/µl)** | 0.09 | 0.19 | 0.58 | 0.08 | 1.19 | 0.09 | 0.05 | 0.09 | 0.11 | 0.04 | 0.1 | 0.12 | 0.75 | 0.01 | 0 | 1.01 | 0.17 |  |  |  |
| **Other CD3+ CD56+ DN (n/µl)** | 0.03 | 0.39 | 3.49 | 0.62 | 6.32 | 2.14 | 0.88 | 0.2 | 1.11 | 0.06 | 2.36 | 0.32 | 0.71 | 0.29 | 1.06 | 0.23 | 1.55 |  |  |  |

*SARS-CoV-2: severe acute respiratory syndrome coronavirus 2; IgG: immunoglobulin G; RU: ratio units; ml: milliliter; n: number; µl: microliter; DP: double positive (CD4+CD8+) cells; DN: double negative (CD4-CD8-) cells; iNKT:invariant natural killer cells; MAIT: mucosal-associated invariant T cells; γδ: gamma-delta T cells; NK: natural killer cells; Other: other than iNKT, MAIT, γδ; Seroconversion: seroconverted:1, not seroconverted:0*

**Supplementary Table 5.** Number of participants (percentage) with each lymphocyte subpopulation value within age-related normal values on the day of the first vaccination.

| **Baseline cellular parameters** |  | **Oncology patient**  n=17 | **Healthy controls**  n=13 |
| --- | --- | --- | --- |
| Total lymphocyte count | n/µl | 9/20 (45%) | 12/13 (92.3%) |
| CD3+ | n/µl | 2/17 (11.8%) | 10/13 (76.9 %) |
| CD4+ | n/µl | 3/17 (17.6%) | 10/13 (76.9 %) |
| CD8+ | n/µl | 5/17 (29.4%) | 11/13 (84.6%) |
| CD4/CD8 ratio |  | 12/17 (70.6%) | 13/13 (100%) |
| CD3+CD56+** | n/µl | 8/17 (47.1%) | 9/13 (69.2%) |

*n:number; µl: microliter; **: age-specific predicted values*

No data on age-related normal values are available for the other lymphocyte subpopulations in Tables 4 and 5.
