# Supplementary figures and images for: Possible role of pre-vaccination T-lymphocyte subpopulations in the antibody response to COVID-19 vaccines in children undergoing chemotherapy
Source: Front Immunol. 2026 Feb 5;17:1728845. doi: 10.3389/fimmu.2026.1728845 (PMC12916594; doi:10.3389/fimmu.2026.1728845)

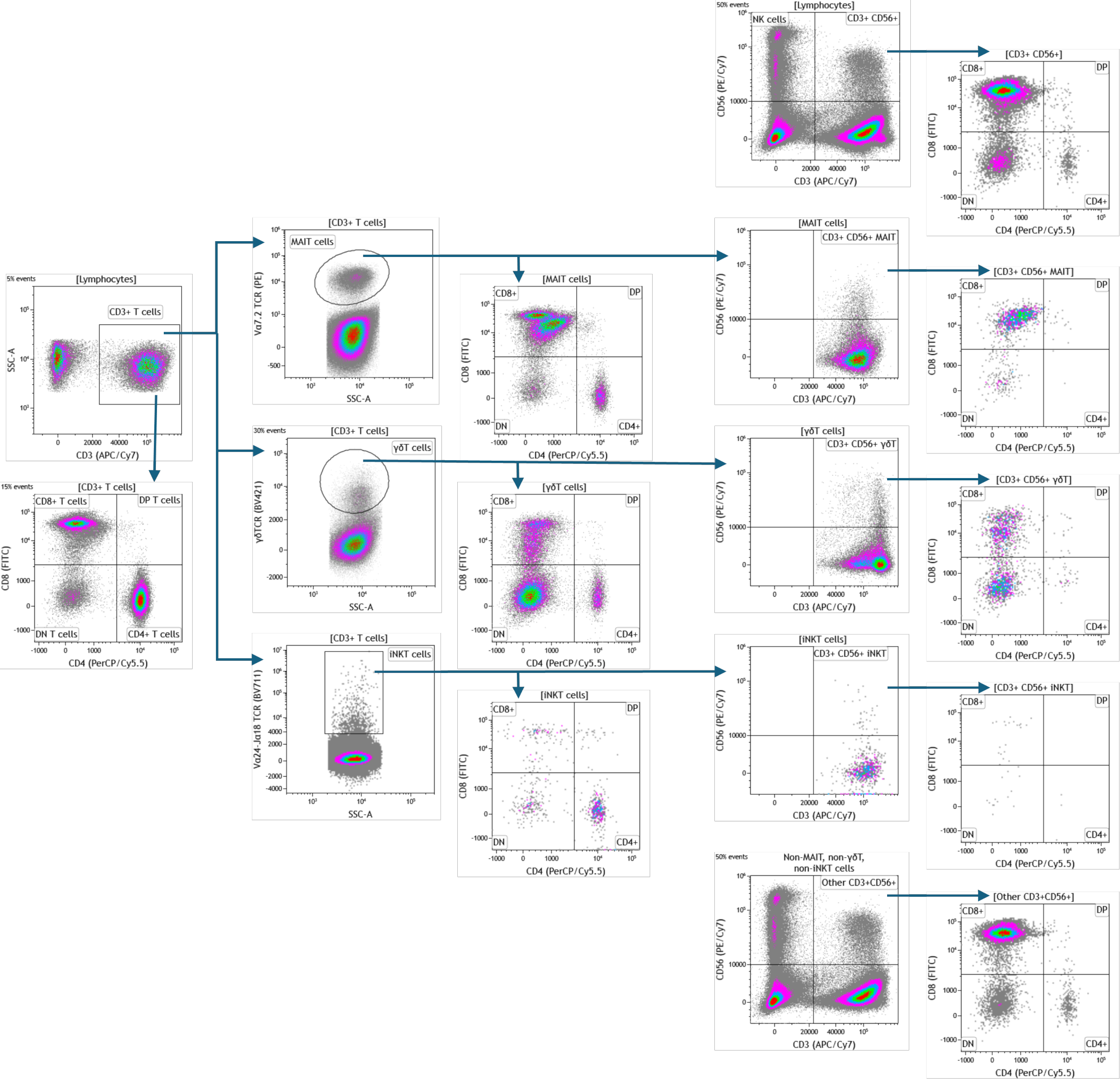

Supplement: Supplementary file 2 [file Image1.tif]
